# Multiple tissue-specific expression of rice seed-shattering gene *SH4* regulated by its promoter pSH4

**DOI:** 10.1186/s12284-015-0047-4

**Published:** 2015-02-14

**Authors:** Huanxin Yan, Li Ma, Zhe Wang, Zhimin Lin, Jun Su, Bao-Rong Lu

**Affiliations:** Ministry of Education Key Laboratory for Biodiversity and Ecological Engineering, Institute of Biodiversity Science, Fudan University, Songhu Road 2005, Shanghai, 200436 China; Fujian Province Key Laboratory of Genetic Engineering for Agriculture, Fujian Academy of Agricultural Sciences, Wusi Road 247, Fuzhou, 350003 China

**Keywords:** *Oryza sativa*, Abscission, Gene expression, Sequence analysis, *cis*-acting elements, GUS, *Agrobacterium*-mediated transformation

## Abstract

**Background:**

Rice seed shattering is an important domestication syndrome encoded by a gene named as *SH4*. The coding region of *SH4* has been well studied regarding its function and roles in evolution. However, its promoter has not been identified, which limited our understanding of the detailed regulatory mechanisms of this gene. It is therefore critical to characterize the promoter and study its expression pattern.

**Results:**

We analyzed the 5′ upstream sequences of this gene and identified a ~2.6 kb fragment with typical promoter features, which was designated as pSH4. The promoter contained a number of *cis*-acting elements related to abscisic acid (ABA) and a CpG island that were characteristics of multiple tissue-specific expression. We isolated and ligated pSH4 to the β-glucuronidase (GUS) reporter gene, and transformed it into a *japonica* rice cultivar to determine the multiple expression pattern of *SH4*. Histochemical location and fluorescence analyses of GUS activity of transgenic plants indicated multiple tissue-specific expression of pSH4 in the seed-pedicel junction region of mature panicles (with highest level), stems, coleoptiles of germinated seeds, and scutella of mature seeds.

**Conclusions:**

The multiple tissue-specific expression pSH4 is categorized as a spatiotemporal promoter that drives the expression of the *SH4* gene in different rice tissues, in addition to the seed-pedicel junction region. Our findings suggest that *SH4* may have additional functions in the growth and development of rice, apart from its major role in seed shattering.

## Background

A promoter is a sequence of DNA that regulates gene transcription. In plant genetic engineering, promoters have been widely used to achieve specific expression of transgenes for crop improvement and gene function analysis (Odell et al. 1985; Nandi et al. 2002). Based on the types of gene expression regulation, promoters are divided into three categories: (i) constitutive promoters that are active constantly in most or all tissues; (ii) spatiotemporal promoters that have development-stage-specific or tissue-specific activity; and (iii) inducible promoters that are regulated by external physical or chemical signals (Ye et al. 2012). The growth and development of plants is controlled by specific expression of certain genes at an appropriate time, in a particular tissue, and at appropriate abundance (Wray et al. 2003). Through interaction with transcription factors, a promoter regulates gene expression at a specific concentration within cells or tissues under particular environmental conditions (Smale 2001). In the last few years, it is popular to discover a gene characterized tissue-specific expression with a special promoter, for analyzing the growth and development of an important trait and applying in plant genetic engineering (Bommert et al. 2005). Many tissue-specific promoters restrictedly expressed in particular cells, tissues, organs, or developmental stages have been identified (Hwang et al. 2002; Gupta et al. 2007). Obviously, the tissue-specific expression of a gene is governed by the combined action of *cis*-acting elements in its promoter region, as well as different nuclear proteins interacting with the elements. Therefore, the *cis*-acting elements function as activators or repressors in regulating leaf-, stem-, root-, and panicle-specific gene expression (Cai et al. 2007).

Abscission is a programmed organ-detachment process, in which plant parts such as fruits, seeds, and leaflets separate from their mother plants in response to developmental cues to guarantee efficient dispersal or propagation of the plants (Patterson 2001). The loss of natural fruit abscission or seeds shatter is one of the key change of agricultural traits in domestication of cereals, as a result of selection by humans (Sang and Ge 2007). Asian cultivated rice (*Oryza sativa* L.) is one of the world most important cereal crops, and shows diverse degrees of seed shattering from relative persistence to easy-shattering in different cultivars (Konishi et al. 2006). Commonly, wild and weedy relatives of rice have a strong seed-shattering ability (Thurber et al. 2011). Seed shattering in rice depends on the proper formation and subsequent degradation of an abscission zone, mostly encompassing one layer of small, thin-walled densely cytoplasmic cells, between seeds and pedicels (Lewis et al. 2006; Thurber et al. 2011). Through genetic analyses of rice cultivars and wild progenitors, several quantitative trait loci (QTL) associated with seed shattering have been identified, including *SH4*, *qSH1*, *OsCPL1*, and *SHAT1* (Li et al. 2006a; Konishi et al. 2006; Ji et al. 2010; Zhou et al. 2012).

Li et al. (2006b) fine-mapped and cloned the *SH4* gene based on the crosses between a rice cultivar CL16 (*O. sativa* subsp. *indica*) and an annual common wild rice line IRGC80470 (*O. nivara*). They considered that the *SH4* gene explained 69% of variation in seed-shattering. A single non-synonymous substitution (G to T) in the Myb3 DNA-binding domain of the gene results in the incomplete development and partial dysfunction of the abscission zone where the separation of a seed from the mother plant occurs, leading to reduced seed shattering of cultivated rice (Li et al. 2006b), although recent studies inferred that this gene may not necessarily play a key role in the early domestication of rice (Thurber et al. 2010; Zhu et al. 2012). The *SH4* gene is the first cloned gene that governs rice seed shattering and is the most significant seed-shattering gene for rice domestication (Doebley 2006).

A previous study identified the specific expression feature of *SH4* mRNA at the seed-pedicel junction that included the abscission zone, and a trend of increased gene expression at the seed maturity (Li et al. 2006b). However, little was known about the regulatory sequence of *SH4* although a previous complementation test implied that a *~*2.6 kb upstream region of *SH4* might have the regulatory function, affecting seed shattering in rice (Li et al. 2006b). Hence, the function of *SH4* promoter, particularly the tissue-specific expression pattern, needs further investigation. To characterize the ~2.6-kb fragment that includes *SH4*’s promoter (Li et al. 2006b) for its *cis*-acting elements, and its expression pattern in different tissues may provide insights into the regulatory mechanisms of seed shattering in rice.

A number of promoters for abscission-related genes has been isolated and their expression pattern was investigated in different plants by the β-glucuronidase (GUS) fusion system (Jefferson et al. 1987), such as oilseed rape and *Arabidopsis* (González-Carranza et al. 2002; Farage-Barhom et al. 2008). Yet, the promoter of seed-shattering genes in rice has not been well studied. With the completion of rice total genome sequencing (Kawahara et al. 2013), it becomes very convenient to study the regulatory region of genes crucial for plant growth and development, including that of seed-shattering genes. Here, we report the isolation of the promoter of rice seed-shattering gene *SH4*, from a *japonica* rice cultivar, Nipponbare. The objectives of this study were to: (i) characterize the ~2.6-kb fragment for its *cis*-acting elements; (ii) examine the tissue-specific expression pattern and activity of the identified promoter. To achieve the objectives, we produced transgenic rice plants containing a chimeric gene construction with the potential promoter region of *SH4* fused with the *GUS* gene, and examined the expression pattern of GUS activity. The generated knowledge will facilitate our understanding of the possible multiple tissue-specific expression of the rice seed-shattering gene *SH4* regulated by its promoter.

## Results

### Identifying a promoter upstream of the rice seed-shattering gene *SH4*

**Figure 1:**
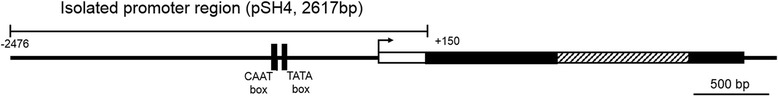
**Structure of** ***SH4*** **gene including a part of its coding region and 5′ upstream region.** White box: the 5′-untranslated region (UTR); black boxes: exons; striped box: intron. The direction and initial of transcription is indicated by an arrow. The vertical bars indicate the putative CAAT box and TATA box. -2476 and +150 indicate the distance of upstream or downstream from the transcription start site.

Based on the genomic DNA sequence of *japonica* cultivar Nipponbare from the rice genome annotation database (RGAR-7), we identified a *~*2.6 kb fragment in the intergenic region upstream of the seed-shattering gene *SH4*. Sequence analysis of this fragment based on the PLACE database indicated the basal regulatory elements for a promoter, TATA-box and CAAT-box that were close to the seed-shattering gene *SH4* at the position −751 and −801, respectively (Figure 1). As usual, the transcriptional start site (TSS) found at 144 bp upstream of translation start-codon ATG of *SH4* was determined as +1, based on the PlantPAN database. The results demonstrated the likelihood of the isolated fragment that had the promoter function. This potential promoter was designated as pSH4. In addition, a CpG island involved in gene transcription was detected at the position from −377 to +150. Multiple *cis*-acting elements were also detected in pSH4 (Table 1). The characteristics of pSH4 suggested its possible function for the tissue-specific expression of *SH4*.

**Table 1:** **The** ***cis*** **-acting elements related to tissue-specific expression identified in pSH4 promoter**

**Name**	**Motif**	**Position**	**Putative regulatory function (references in parentheses)**
TRAB1	CAACGTGTGAC	−1775	Binding site for ABA signal transaction gene *TRAB1* (Hobo et al. 1999).
OSBZ8	ACGTGTGCTCCATC	−963	Binding site for ABA signal transaction gene *OSBZ8* (Nakagawa et al. 1996).
ACGTATERD1	ACGT	−1773, −1435, −1037, −961, −846, −819, −679	ABRE required for dehydration stress and dark-induced senescence (Simpson et al. 2003).
DPBFCOREDCDC3	ACACNNG	−726, −13	Binding core sequence found in the carrot embryo-specific *Dc3* gene promoter, and induced by ABA (Kim et al. 1997).
RYREPEATVFLEB4	CATGCATG	−429	RY repeat motif, related to ABA-regulated gene expression during late embryo-genesis (Hobo et al. 1999).
MYB1AT	WAACCA	−1345, −1201, −75	MYB recognition site for dehydration-responsive gene and mediated by ABA (Abe et al. 2003).
MYCATERD1	CATGTG	−1252, −1221	MYC recognition sequence for early responsive to dehydration and mediated by ABA (Simpson et al. 2003).
MYCCONSENSUSAT	CANNTG	−1601, −1424, −1415, −1369, −1339, −1252, −1221, −435	MYC recognition site found in the promoters of the dehydration-responsive gene and mediated by ABA (Abe et al. 2003)
MYBGAHV	TAACAAA	−693	GARC involved in gibberellin signal pathway and sugar suppression (Gubler et al. 1995).
WRKY71OS	TGAC	−2062, −1934, −1845, −1768, −1755, −869, −717	A core of W-box, involved in gibberellin and ABA signaling pathways (Zhang et al. 2004).
AMYBOX1	TAACARA	−693	Amylase box, conserved sequence found in the promoter of α-amylase gene, regulating specific expression in germinating seeds and callus (Hwang et al. 1998).
AMYBOX2	TATCCAT	−1794	Amylase box, conserved sequence found in the promoter of α-amylase gene, regulating specific expression in germinating seeds and callus (Hwang et al. 1998).
GATABOX	GATA	−1887, −1479, −1236, −813, −503	Conserved in light-regulated and tissue-specific expression genes (Lam and Chua 1989).

N = Any base; R = G or A; Y= C or T; W = A or T.

### Obtaining transgenic rice plants containing pSH4-GUS

**Figure 2:**
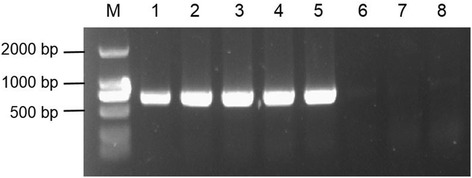
**Results of PCR products amplified by gusAF/gusAR primers for identifying transgenic plants.** The expected size of PCR products is 662 bp. Track 1: 35S-GUS plant (positive control); track 2–6: pSH4-GUS transgenic plants; track 7: Nipponbare (negative control); track 8: water (blank control). M: D2000 DNA ladder.

**Figure 3:**
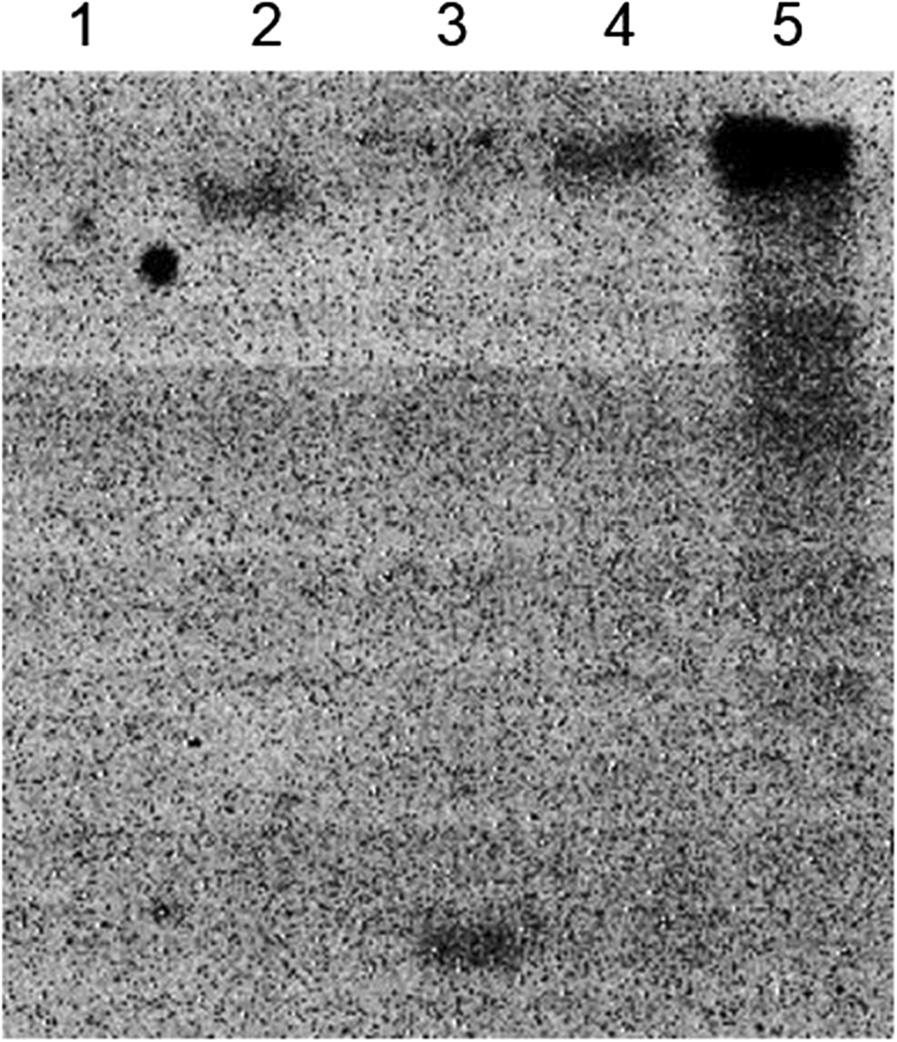
**Southern blot analysis of pSH4-GUS transgenic plants.** Track 1: Nipponbare (negative control); track 2–4: pSH4-GUS transgenic lines (T-37, T-25, and T-20, respectively); track 5: pSH4-GUS plasmid (positive control).

The pSH4 fragment with 2,617-bp was isolated from the genome of Nipponbare. The recombinant binary vector, pSH4-GUS was constructed and transformed into Nipponbare. More than 50 transgenic plants (T_0_) were obtained. Of these transgenic plants, five well-developed plants were selected and subjected to produce later generations (T_1–3_) through self-pollination. After molecular confirmation with PCR identification (Figure 2) and Southern blot analysis (Figure 3), three transgenic plant lines (T-20, T-25 and T-37) containing single-copy and homozygous transgene (pSH4) were selected for further analysis to test the function of the promoter for its tissue-specific expression.

### Tissue-specific expression patterns of the promoter pSH4

**Figure 4:**
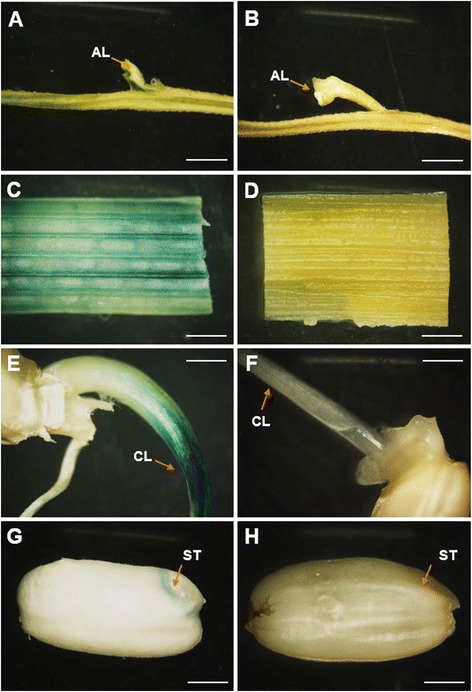
**GUS histochemical staining results in different tissues driven by pSH4.** GUS-staining (blue dye) of different tissues in pSH4-GUS transgenic plants **(A, C, E, and G)** and non-transgenic controls **(B, D, F, and H)**. **A** and **B**: branches of panicle, showing the seed-pedicel junction region (AL, indicated by arrows); **C** and **D**: stems (internodes); **E** and **F**: coleoptiles (CL, indicated by arrows) from germinated seeds; **G** and **H**: Dehusked mature seeds, showing scutella (ST, indicated by arrows). Bar = 1mm.

Our results showed the tissue-specific expression of GUS regulated by pSH4 in T_2_-T_3_ transgenic plants (Figure 4A-H). GUS staining was observed in the seed-pedicel junction region (where seeds separated from mother plants) of mature panicles (30 DAF) in the histochemical stained pSH4-GUS transgenic plants (T_2_, Figure 4A). These results confirmed pSH4 was the promoter of the seed-shattering gene *SH4*. Similarly, GUS staining was also detected in stems (T_2_, Figure 4C) and coleoptile of germinated seeds (T_3_, Figure 4E). However, only weak GUS staining was observed in the scutella of embryos of mature seeds, although no staining was found in endosperms (T_3_, Figure 4G). GUS staining was not observed in roots, leaves, and flowers at different developmental stages of the transgenic plants. Differently, GUS staining was observed in all tissues (including roots, stems, leaves, panicles and seeds) of the positive control, 35S-GUS transgenic plants (data not shown), suggesting the effectiveness of GUS staining. In contrast, no GUS staining was observed in all corresponding tissues of the negative control, non-transgenic Nipponbare (Figure 4B, D, F, and H).

**Figure 5:**
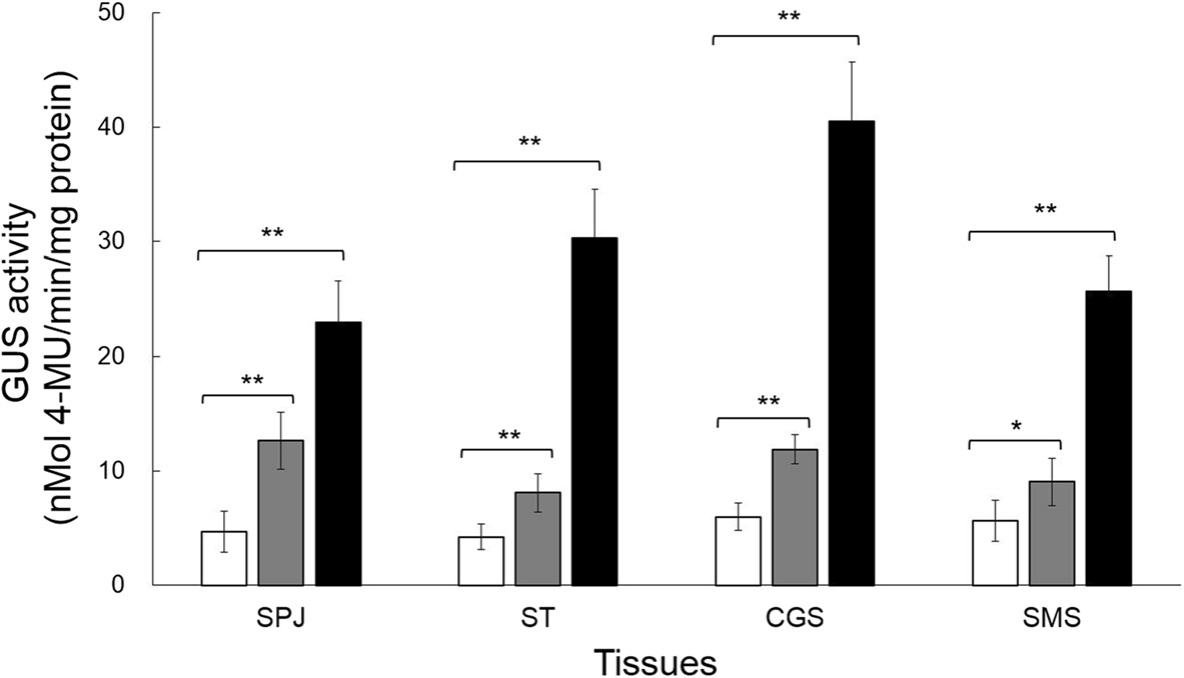
**GUS fluorescent assay in different tissues of pSH4-GUS and 35S-GUS transgenic plants.** SPJ: seed-pedicel junction region of panicles; ST: stems; CGS: coleoptiles of germinated seeds; SMS: scutella of mature seeds. Differences were compared between non-transgenic control (white columns) and pSH4-GUS transgenic lines (gray columns) or 35S-GUS transgenic plants (black columns), respectively, using the independent *t*-test. *, P<0.05; **, P<0.01. Bars indicate standard errors.

In addition, our results demonstrated the lower expression of pSH4 than that of 35S promoter in tested tissues of the transgenic plants. This was reflected from the GUS quantitative assays that showed a significantly reduced GUS staining level (to 27%-55%) in different tissues of pSH4-GUS transgenic plants (Figure 5). GUS expression level in the seed-pedicel junction region of pSH4-GUS transgenic plants was only an average of ~55% that of 35S-GUS transgenic plants. Even more reduced GUS staining levels were detected in stems, coleoptile of germinated seeds, and mature embryos (to ~27%, ~29% and ~35%, respectively) (Figure 5). The results suggested the moderate expression strength of pSH4 although with the highest level in the seed-pedicel junction region that was associated with seed shattering.

## Discussion

### *SH4* promoter and its multiple *cis*-acting elements

Based on the study of Li et al. (2006b) who suggested that a 2,640 bp fragment at the 5′ upstream of the *SH4* gene had the function of regulating the expression of *SH4*, we isolated a 2.6-kb fragment, ranging from the position −2,472 to +150, to study its multiple tissue-specific function as a promoter. Sequence characterization involving the PLACE and PlantPAN databases showed the basal regulatory elements: the TATA-box and CAAT-box that are typical elements for promoter function. Thus, this identified promoter was named as pSH4. In addition, a CpG island that is considered to be related to the tissue-specific expression of a gene was also identified as a part of pSH4. It is known that a gene regulated by a promoter usually has multiple expression in two or more tissues, if a CpG island presents in the 5′ end of the promoter (Ashikawa 2002). Given that the presence of a CpG island in pSH4 is located at its 5′ end, this promoter is therefore expected to have the function to regulate the *SH4* gene expression in multiple tissues. Previous studies of *SH4* mostly focused on its gene-coding region (Zhang et al. 2009; Thurber et al. 2010). However, knowledge on the regulatory mechanism of this gene is limited due to its unidentified promoter. The cloning of pSH4 can largely expand the studies from the *SH4* sequence and its function to the regulatory mechanisms associated with this promoter. Further studies of the pSH4 polymorphisms of this domestication-related gene may also increase our understanding of the adaptive evolution of seed shattering in cultivated, weedy, and wild rice samples (Zhu et al. 2012).

In addition, multiple *cis*-acting elements were identified in pSH4 in this study. Usually, the type, number, and position of *cis*-acting elements within a promoter region can determine the spatiotemporal expression of the gene regulated by a promoter (Cai et al. 2007). In this study, different types of the *cis*-acting elements that are associated with tissue-specific gene expression were identified in pSH4. For example, the abscisic acid response elements (ABREs) that are involved in the abscisic acid (ABA) hormone signal pathways were identified (Table 1). These findings indicate that pSH4 is most likely associated with the formation and activity of the abscission zone, which depends on the presence of ABA (González-Carranza et al. 1998; Li et al. 2006b). The cloning of pSH4 provides opportunities to investigate such regulatory mechanisms of ABA hormone signal pathway in relation to the abscission zone that is closely associated with seed shattering in rice.

### Multiple tissue-specific expression pattern of pSH4

The GUS expression in this study revealed both by histochemical and fluorescence assays in pSH4-GUS transgenic plants indicated the multiple tissue-specific expression pattern regulated by pSH4, as expected from the characterization of this promoter. Therefore, the pSH4 can be categorized as a spatiotemporal promoter. This result suggested the additional function of *SH4* besides its important role in seed shattering. The major function of *SH4* concerning seed shattering has been illustrated by the analyses of RT-PCR and *in-situ* hybridization in the seed-pedicel junction region (Li et al. 2006b, Zhou et al. 2012). We reconfirmed in this study that the GUS reporter gene driven by pSH4 had the highest level (~55%) of expression in the seed-pedicel junction region of mature panicles, relative to that of driven by 35S promoter. Given that the seed-pedicel junction region includes the abscission zone (Thurber et al. 2011), the role of *SH4* in controlling seed shattering is probably related to the formation and activity of the abscission zone during seed ripening. Detailed studies involving pSH4 may completely reveal the regulatory mechanisms of *SH4* in relation to the abscission-related traits in rice such as seed shattering. The promoter regulated gene expression in the abscission zone can also serve as a bridge to uncover new genes involved in abscission process (González-Carranza et al. 2012). In the case of practical uses, the sequence of a seed-shattering promoter such as pSH4 can be manipulated as the target of TALEN-based gene editing for plant engineering (Li et al. 2012). For example, genetically engineered rice with reduced seed shattering by TALEN technique may mitigate the impact of transgene flow from transgenic rice to weedy rice on the seed dispersal of this weed (Gressel and Valverde 2009).

In addition to the seed-pedicel junction region of mature panicles, the GUS activity driven by pSH4 was also observed in other tissues of stems, coleoptiles of germinated seeds and scutella of mature seeds with a relative low level of expression. To the best of our knowledge, the expression of *SH4* in these tissues has never been reported before. However, the function of *SH4* expression in these tissues remains unknown. Interestingly, the expression pattern coincides with a number of *cis*-acting elements presented in pSH4. For example, a group of ABREs closely associated with the ABA signal pathways, including two binding sites for ABA signal transaction genes were identified (see Table 1). In addition to its association with abscission, ABA signaling can activate the expression of genes in seeds and other vegetative tissues when they become dehydrated (Finkelstein et al. 2002). In addition, ABA can also inhibit the elongation of stems and induce transition from seed maturation/dormancy to germination (Finkelstein et al. 2002; Krouk et al. 2011). Altogether, we expect that the function of *SH4* might be associated with ABA signaling during the growth and development of rice plants at different stages, such as seed germination, stem development, and seed ripening, at which the outcome of *SH4* expression leads to seed shattering of weedy and wild rice.

Furthermore, the expression pattern of pSH4 in the early stages of rice seed germination and seedling development associated with the AMYBOX elements found in pSH4 is similar to that of the rice α-amylase and sucrose synthase genes, contributing to the mobilization of carbon from source to sink during seed germination and the subsequent stages of seedling development regulated by sugar and hormone signals (Chávez-Bárcenas et al. 2000; Chen et al. 2006). The linkage between our findings and the case of α-amylase and sucrose synthase genes indicates the similar function of *SH4* in hormone signal pathways.

## Conclusions

Sequence analyses and GUS activity determination demonstrated the multiple-tissues expression of pSH4 in the seed-pedicel junction region, stems, coleoptiles of germinated seeds, and scutella of mature seeds. This tissue-specific expression pSH4 is categorized as a spatiotemporal promoter. The multiple tissue-specific expression pattern of pSH4 indicates that *SH4* may play a diverse role in the growth and development in rice, in addition to seed shattering. Further investigation on the expression profile of *SH4*, together with its upstream *cis*-acting elements in pSH4 will provide deeper insights into the mechanisms of its function in rice seed shattering, in addition to its potential roles in seed germination/dormancy and seedling growth associated with hormone signals. Full understanding of the mechanisms of seed-shattering related genes, including *SH4*, will help to uncover the domestication processes in rice, where seed shattering plays a key role.

## Methods

### Identifying and isolating the promoter of *SH4*

The sequences of an intergenic region in the 5′ upstream of *SH4* (LOC_Os04g57530.1) with physical location between 34,233,221~34,235,832 base pairs (bp) on chromosome 4 of a *japonica* rice cultivar Nipponbare were downloaded from Rice Genome Annotation Release 7 (RGAR-7) at the Michigan State University (MSU) (Kawahara et al. 2013) for promoter analysis. The transcriptional start site of *SH4* and CpG island in the upstream region of *SH4* were predicted using the PlantPAN database (Plant Promoter Analysis Navigator; Chang et al. 2008). The *cis*-acting elements of the promoter were determined based on the PLACE (Plant *Cis*-acting Regulatory DNA Elements; Higo et al. 1999) and PlantPAN databases.

For isolating the predicted promoter of *SH4* (from position −2467 to +150), genomic DNA was extracted from young leaf tissues of Nipponbare seedling, using the CTAB method (Doyle 1987). Based on the 5′ upstream genomic sequences of *SH4*, a primer pair (psh4F-xba: 5′-AGAGGC*TCTAGA*TTCGATTCCC-3′ and psh4R-bgl: 5′-GCAGAG*AGATCT*GACATGCTCG-3′) was designed (the underlined letters indicated XbaI/BglII restriction sites) to facilitate directional cloning of the promoter.

PCR amplification was carried out by initial denaturation at 94°C for 4 min followed by 35 cycles of 94°C denaturation for 30 s, 55°C annealing for 30 s, and 72°C elongation for 40 s, and with a final extension of 72°C for 10 min. The 50 μl reaction mixture for the PCR consisted of an aliquot of 50 ng template DNA, 1.5 mM MgCl_2_, 0.2 mM each of dNTP, 1 unit of Taq DNA polymeraseand 10 pmol each of the primers. PCR products were separated in 1% (w/v) agarose gel and purified using the gel extraction kit.

The PCR products were cloned into the pMD18-T vector (TaKaRa Inc., Dalian, China) by T4 DNA polymerase, and named as pMD18-pSH4. Then the plasmid was introduced into *Escherichia coli* strain DH5α with heat shock method (Sambrook et al. 1989). The plasmids of bacteria were extracted by the plasmid extraction kit and confirmed by sequencing using two primers (M13F: 5′-TGTAAAACGACGGCCAGT-3′ and M13R: 5′-CAGGAAACAGCTATGACC-3′). Sequence homologies were determined by local alignments with SeqMan 5.01 software (DNASTAR Inc.).

### Constructing pSH4-GUS vector and producing transgenic rice plants

**Figure 6:**
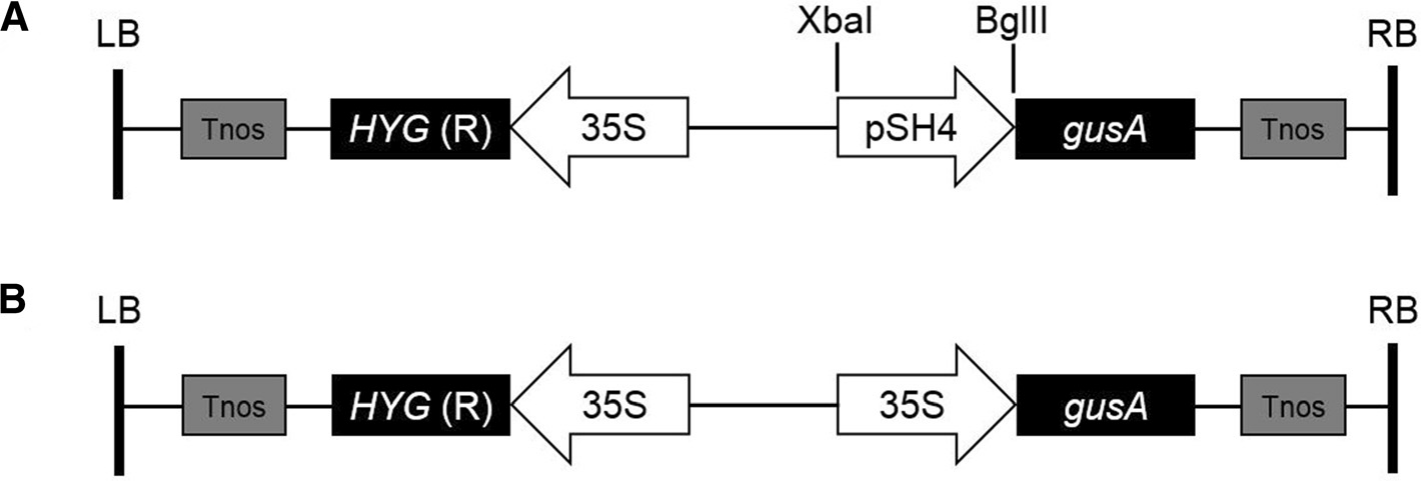
**Structure of pSH4-GUS (A) and 35S-GUS (B) constructs for rice (Nipponbare) transformation.** Tnos: terminator of nopaline synthetase; *HYG*(R): hygromycin-resistence gene; 35S: cauliflower mosaic virus (CaMV) 35S promoter; pSH4: SH4 promoter; XbaI and BglII indicate restriction sites; *gusA*: β-glucuronidase (GUS) gene; RB: right border; LB: left border.

The isolated fragment of potential promoter in the vector pMD18-pSH4 was inserted into the XbaI/BglII sites of vector pCAMBIA1301 to replace the CaMV35S promoter upstream from the GUS reporter gene to generate a recombinant vector, pSH4-GUS (Figure 6A). The inserted fragment was confirmed by PCR amplification with a primer pair (psh4aF: 5′-GCTGATCCGCTGGCCGTAGAAGTC-3′ and psh4aR: 5′-GCGCGTGAAGGGAGGGGGTTTA-3′) and sequenced with two primers (Pseq1: 5′-CCAGGCTTTACACTTTATGC-3′ and Pseq2: 5′-TTCACGGGTTGGGGTTTCTAC-3′). The pCAMBIA1301 vector without any inserts, where the GUS gene was still driven by a constitutive promoter CaMV35S, was used as the positive control for GUS analysis, and named as 35S-GUS (Figure 6B).

The plasmids of the recombinant vectors, pSH4-GUS and 35S-GUS were both introduced into *Agrobacterium tumefaciens* strain LBA4404 (Hoekema et al. 1983) by electroporation (Sambrook et al. 1989), and transformed into rice calli induced from mature embryos of Nipponbare (Hiei et al. 1994). Regenerated plants (T_0_ generation) were eventually transferred to soil in pots and grown to maturity in a greenhouse.

### Cultivation and identification of transgenic rice

Transgenic and non-transgenic rice seeds were germinated in the Petri-dish with moist filter papers at 37°C for 2 days in dark. Germinated seeds were grown in pots with soil in an illuminated climate cabinet at 30°C. After 30 days, well-grown seedlings were transplanted into a paddy field filled with rice nursery soil. Transgenic plants of T_1_-T_3_ generations were produced from self-pollination of T_0_ transgenic individuals.

To identify transgene-positive plants, the transgenic plants were examined by PCR with the primer pair (gusAF: 5′-ACGACTCGTCCGTCCTGTAG-3′ and gusAR: 5′-CCGCATCACGCAGTTCAA-3′). To determine the copy number of the transgene, Southern blot analysis was carried out. Fresh leaves of the transgenic (T_2_ generation) and non-transgenic control plants were used to extract total genomic DNA (Murray and Thompson 1980) and quantified spectrophotometrically. About 30 μg of genomic DNA was digested with BamHI restriction enzyme, size-separated in a 1% (w/v) agarose gel and transferred onto the Hybond-N+ nylon membrane (Amersham Pharmacia, Uppsala, Sweden) by a capillary transfer procedure. DNA hybridization was carried out following the method by Sambrook et al. (1989) using a *HYG* (R) gene-specific probe and imaged.

### Determining histochemical and fluorescent GUS expression

Tissue samples from at least three pSH4 transgenic lines (T_2_-T_3_ generations), the 35S-GUS transgenic and non-transgenic control plants were included for GUS analyses, including its expression profiles and quantitative assay. Leaf (blade) and stem (internode) tissues were collected at the growth stages between two months and three months after seed germination. Flower- or seed-pedicel junction regions, containing 1 mm of a pedicel and 1.5 mm of the attached flowers or seeds from young panicles, were sampled at 15 days after flowering (DAF), and mature panicles were sampled at 30 DAF (Li et al. 2006b). These samples were collected from the T_2_ plants. Embryos and endosperms of mature seeds, plumules (including coleoptiles) and radicles of seedlings five-day after seed germination, and young leaves and roots of seedlings about 20 days after seed germination were collected from its T_3_ plants.

GUS expression profiles were determined by histochemical assay (Jefferson 1987). The collected tissue samples were immersed in GUS Staining Buffer (100 mM sodium phosphate buffer pH 7.0, 0.5 mM K_3_[Fe(CN)_6_], 0.5 mM K_4_[Fe(CN)_6_], 10 mM Na_2_EDTA, 0.1% (v/v) Triton X-100) supplemented with 1 mM X-Gluc solution, and incubated at 37°C for overnight (*c.* 24 h) in the dark. After bleached with ethanol to remove chlorophyll, the stained samples were observed and photographed under a dissecting microscope.

GUS quantitative assay was performed using the fluorescent method described by Jefferson (1987). The collected tissue samples were homogenized in a GUS Extraction Buffer (50 mM Na_2_HPO_4_ at pH 7.0, 10 mM EDTA, 0.1% sodium laurylsarcosine, 0.1% (v/v) Triton X-100 and 10 mM β-mercaptoethanol). Total protein concentration of the samples was determined by the Bradford assay method (Bradford 1976). GUS activity was determined with a fluorescence photometer by measuring the amount of 4-methylumbelliferone (4-Mu) produced under the catalysis of GUS in 1 milligram of total protein per minute. The assay for each sample was repeated three times. Six biological from the three independent pSH4-GUS transgenic lines (T-20, T-25, and T-37), the 35S-GUS transgenic plants and non-transgenic control of each tissue sample were performed.

The expression level of the promoters were calculated as the differences between the mean values of GUS quantitative assay results of pSH4-GUS or 35S-GUS transgenic plants and non-transgenic controls. The relative promoter expression strength of pSH4 was determined by the ratio of GUS activity driven by pSH4 and 35S promoters. Significant differences between transgenic and non-transgenic plants were determined by the independent *t*-test using the software package SPSS ver. 19.0 for Windows (IBM Inc., New York, USA).

## Abbreviations

4-Mu: 4-methylumbelliferone
ABA: Abscisic acid
ABRE: Abscisic acid response element
CaMV: Cauliflower Mosaic Virus
DAF: Day(s) after flowering
*HYG* (R): Hygromycin-resistance gene
GUS: β-glucuronidase
PLACE: Database of Plant *Cis*-acting Regulatory DNA Elements
PlantPAN: Database of Plant Promoter Analysis Navigator
RGAR-7: Rice genome annotation database release 7
TALEN: Transcription activator-like (TAL) effector nuclease
TSS: Transcriptional start site
